# Testing experiences of HIV positive refugees in Nakivale Refugee Settlement in Uganda: informing interventions to encourage priority shifting

**DOI:** 10.1186/1752-1505-7-2

**Published:** 2013-02-14

**Authors:** Kelli N O’Laughlin, Shada A Rouhani, Zikama M Faustin, Norma C Ware

**Affiliations:** 1 Brigham & Women’s Hospital, 75 Francis Street, Boston, Massachusetts, 02115, USA; 2 Harvard Medical School, 641 Huntington Avenue, Boston, Massachusetts, 02115, USA; 3 Massachusetts General Hospital, 75 Francis Street, Boston, Massachusetts, 02115, USA; 4 Bugema University, PO Box 6529, Kampala, Uganda, USA

**Keywords:** HIV testing, Refugees, HIV, HIV/AIDS, Qualitative, Sub-Saharan Africa, Uganda, Africa

## Abstract

**Background:**

Recent initiatives by international health and humanitarian aid organizations have focused increased attention on making HIV testing services more widely available to vulnerable populations. To realize potential health benefits from new services, they must be utilized. This research addresses the question of how utilization of testing services might be encouraged and increased for refugees displaced by conflict, to make better use of existing resources.

**Methods:**

Open-ended interviews were conducted with HIV-infected refugees (N=73) who had tested for HIV and with HIV clinic staff (N=4) in Nakivale Refugee Settlement in southwest Uganda. Interviews focused on accessibility of HIV/AIDS-related testing and care and perspectives on how to improve utilization of testing services. Data collection took place at the Nakivale HIV/AIDS Clinic from March to July of 2011. An inductive approach to data analysis was used to identify factors related to utilization.

**Results:**

In general, interviewees report focusing daily effort on tasks aimed at meeting survival needs. HIV testing is not prioritized over these responsibilities. Under some circumstances, however, HIV testing occurs. This happens when: (a) circumstances realign to trigger a temporary shift in priorities away from daily survival-related tasks; (b) survival needs are temporarily met; and/or (c) conditions shift to alleviate barriers to HIV testing.

**Conclusion:**

HIV testing services provided for refugees must be not just available, but also utilized. Understanding what makes HIV testing possible for refugees who have tested can inform interventions to increase testing in this population. Intervening by encouraging priority shifts toward HIV testing, by helping ensure survival needs are met, and by eliminating barriers to testing, may result in refugees making better use of existing testing services.

## Background

Sub-Saharan Africa is home to 68% of the global population living with HIV/AIDS, and to two million refugees displaced by conflict [1-3]. Given the vulnerabilities of refugees to HIV-infection [4-6], an average displacement period of seventeen years [7,8], and the reality that diagnosing and treating HIV/AIDS helps decrease disease transmission [9], the World Health Organization (WHO), the Joint United Nations Programme on HIV/AIDS (UNAIDS), and the United Nations High Commissioner for Refugees (UNHCR), have set goals to scale up HIV testing and comprehensive services for all people, including vulnerable populations facing humanitarian crisis [10-15]. In addition, the Inter-Agency Standing Committee has published guidelines recognizing that HIV prevention, treatment, care and support are essential parts of preparing for and responding to crisis and are services that can be provided in humanitarian settings [16]. Refugees are a unique population requiring focused efforts to ensure appropriate programs are designed to enhance uptake of testing for HIV/AIDS [17-19].

Widespread programmatic response has resulted in increased availability of HIV testing and care services for refugees. UNHCR data show that, despite regional variations, many internally displaced peoples and refugees now have access to HIV testing and other comprehensive services [20]. Behavioral surveillance surveys conducted by UNHCR in Kenya, Tanzania and Uganda in 2004/2005 and again in 2010, show that the percentage of participants who have had an HIV test and received results in the previous 12 months increased considerably over the study period [21].

While limited data is available on refugee-specific barriers to HIV testing, many have aimed to characterize barriers to testing for low-resource populations in Africa. Pertinent barriers to testing for this population include: the fear of knowing one’s own status [22-26], low awareness of HIV and lack of perceived risk of disease [23,25,27,28], HIV related stigma [22,24,27-30], and lack of access to testing clinics [31]. Proposed interventions to address these barriers have been to provide safe access to testing and HIV-related services [32] and to make testing more accessible through mobile or door-to-door testing [33-35].

Nakivale Refugee Settlement in southwest Uganda is one site committed to providing refugee-centered HIV testing and clinical care. Established in 1960 to accommodate Rwandan refugees, Nakivale now spans 28 square miles and hosts 56,000 refugees. Preventing Mother-to-child Transmission of HIV (PMTCT) services, including HIV testing, medical therapy and counseling, are a part of care provided by the antenatal clinic. HIV testing, in the form of voluntary counseling and testing, has been available at the Medical Teams International Clinic (previously run by Deutsche Gesellschaft für Internationale Zusammenarbeit) since the clinic began in 2005. Provider initiated testing and counseling (PITC) was implemented at the Clinic in July of 2011. HIV testing at the clinic is free and uses serial rapid HIV tests as recommended by the Uganda HIV Rapid Test Algorithm [36]. Results are provided the same day as testing. To enhance accessibility of HIV therapy for those living in Nakivale, distribution of anti-retroviral therapy (ART) at the Clinic began in October of 2008. ART eligibility was previously defined using Uganda national guidelines [36], and now is based on more recent WHO guidelines [37]. HIV-positive individuals who do not qualify for ART based on disease stage or CD-4 count, receive frequent clinical care and regular evaluation to discern if initiation of ART is indicated.

While there are no published HIV prevalence studies from within Nakivale, in the south-western region of Uganda were Nakivale is located, the HIV prevalence is 7.3 percent (8.1% for women, 6.3% for men) [39]. The HIV prevalence rates from the refugees’ countries of origin range from 1.1% - 6.3% [40]. The majority of refugees in Nakivale are from the Democratic Republic of the Congo, where the current HIV prevalence is unknown but is thought to be high because of the use of sexual violence as a weapon of war [41]. In Nakivale, 659 people (1.2% of people in Nakivale) living with HIV/ AIDS were enrolled in care at the single HIV clinic in the settlement at the time of this study. While not everyone infected is eligible for ART, and people who do not feel sick or who are not eligible for ART may not seek care, it is probable that a substantial number of refugees have not utilized the free HIV testing services provided in the settlement and are unaware of their HIV status.

Enhanced availability of HIV testing services for refugees has resulted in more people being tested, yet to derive maximum benefit from existing testing services, utilization of these services can and should be increased. This paper provides information from a qualitative study to inform development of interventions to improve HIV testing service utilization in refugee settlements. The study elicited experiences of refugees in Nakivale who had tested, to identify and describe the circumstances that made testing possible.

## Methods

### Setting

The study took place at the Deutsche Gesellschaft für Internationale Zusammenarbeit (GIZ) Clinic (now the Medical Teams International Clinic) in Nakivale Refugee Settlement in southwestern Uganda. In Nakivale, currently 57% of the population is from the Democratic Republic of the Congo, 22% is from Rwanda, 13% is from Somalia, 6% is from Burundi and the remaining 2% is from Eritrea, Ethiopia, Sudan, Kenya, and Liberia [38]. GIZ is a German governmental organization that ran the health clinic which provided nearly all HIV screening and ART distribution during the study period.

### Sampling and recruitment

These data are part of a larger qualitative study with multiple goals related to HIV testing and treatment among refugees living in Nakivale. Therefore inclusion criteria were: (1) received ART from the GIZ HIV/AIDS Clinic in Nakivale during the five-month data collection period, (2) age ≥ 18 years and (3) willingness and ability to give informed consent. Seventy-three patients were invited to participate in the study and all accepted. All five staff members employed in the clinic during the study period were also invited to participate and four agreed. Data were collected from March to July of 2011.

This study was approved by the Partners Human Research Committee (Boston, Massachusetts, United States of America) and by the Ugandan National Council of Science and Technology (Kampala, Uganda). Written informed consent was obtained from all participants in English, Kiswahili or Kinyarwanda.

### Data collection

Data were collected through in-person qualitative interviews. A local research assistant, a refugee from the Democratic Republic of the Congo, conducted all of the interviews. The research assistant was trained in qualitative data collection techniques by local staff that had training and experience in qualitative research. Interviews, conducted at the GIZ Clinic in a private room, were minimally structured to cover core topics rather than specific questions. This approach ensured consistency in the interview process, while allowing for unanticipated material to surface. Core topics for the patient and clinic staff interviews focused on experiences accessing HIV/AIDS-related testing and care and perspectives on how to improve utilization of testing services. Interviews were conducted in English, Kinyarwanda, or Kiswahili. Patients participated voluntarily and did not receive a stipend.

Interviews were audio-recorded with permission and averaged 50 minutes. After each interview was complete, the research assistant produced a detailed write-up in English using the audio recording and written notes. The interviews were typed as “stream-lined” transcripts; verbatim accounts that included interviewer questions and interviewee responses and excluded non-essential content such as repetition phrases and hesitations. This transcription approach preserves accuracy and captures needed detail while conserving resources. The transcripts were produced directly into English.

### Data analysis

Analysis was inductive and was informed by a grounded theory approach to category construction [39,40], stopping short of offering a definitive theory but instead aiming to provide a framework to explain the data. Descriptive categories related to circumstances leading to HIV testing utilization were organized interpretively to propose an explanation. Interview content suggesting antecedents of successful testing and underuse of HIV testing was retrieved from the data by two separate analysts. Retrieved sections of the text were grouped according to content similarity. These sections of text were then reread to characterize various forms of facilitators and barriers to HIV testing experienced by the refugees. Where discrepancies were found, they were discussed until consensus was reached. The data were reorganized in terms of these characterizations to produce an initial group of categories. Each category forming the group was named, defined, and illustrated through excerpts from the interviews. The category groups were revisited through successive returns to the data in which additional sections of relevant text were extracted. Lastly, categories were grouped to form a narrative of experiences accessing and utilizing HIV testing and care services in this context and a framework for interpreting those experiences.

## Results

Of 659 displaced people known to be living with HIV in Nakivale at initiation of data collection, 83 were receiving ART from the GIZ Clinic. Seventy-seven interviews (73 patients, 4 clinic staff) were completed. Due to equipment loss prior to back-up of data, the final data set consists of sixty-five write-ups or written accounts of interviews (61 patients, 4 clinic staff). Sixty-three were complete transcripts produced from audio recordings and two were write-ups of notes taken on interviews where the interviewee declined audio recording. To the best of our knowledge, there were no remarkable differences in the content of the lost data. There was convergence in themes between the views of the refugees and the health care providers interviewed.

### Study participants

#### Patients

The average age of the 61 patient participants was 40. The majority of participants were female and nearly three-fourths were from Rwanda. Participants had lived in Nakivale for an average of nine years and had an average of 4.5 years of education. Most were married and two of the married participants were separated from their spouse because of war. The majority of participants were Christian (Table 1).

**Table 1:** Patient participant demographics (N=61)

**Average age (in years)**	**40 years**
% Female	59%
Country of origin	Rwanda 74% (N=45)
	D.R.C. 18% (N=11)
	Burundi 7% (N=4)
	Sudan <2% (N=1)
Years in Nakivale (average)	9
Years of education	4.5
Marital status	Married 64% (N=39)
	Widowed 23% (N=14)
	Divorced 11% (N=7)
	Single <2% (N=1)
Religion	Christian 82% (N=50)
	Muslim 16% (N=10)
	Jehovah’s Witness <2% (N=1)
Average travel time to clinic (one way)	2 hours
Average cost to reach clinic (one way)	3,500 Ugandan Schillings (approx $1.50 USD)

#### Clinic staff

Five clinic staff members worked in the HIV GIZ Clinic in Nakivale during study enrollment and four participated in the study. The clinic staff participants averaged 27 years of age and half were female. They were all Ugandan citizens and their average number of years of education was 16. Three were Christian and one was Muslim.

### Qualitative results

Refugees focus the majority of their daily effort on survival. Survival requirements take precedence over HIV testing. Priorities shift under some circumstances, however. Daily life survival priorities and the types of circumstances under which priorities shift in favor of HIV testing are described in the sections below. Implications of this analytic framework for interventions to increase utilization of HIV testing services are also discussed.

### Priorities: food, shelter & safety

Faced with harsh living conditions, refugees’ daily work focuses on obtaining food, repairing shelter and remaining safe. Refugees attending clinic described feeling constantly hungry and having a difficult time accessing food. Clinic days presented particular hardship, as participants often endured long trips and spent considerable amounts of money to reach the clinic. Missing work to attend clinic caused significant stress as it resulted in less food for their family. When asked if he had ever missed a clinic visit, one patient replied, *"Only one day when I went to work so that my children could get what to eat and I was not able to go for drugs. I came back when I was two days late and explained that I had gone to look for food."* Patients spoke of lack of food as a barrier to overall health. Some described the difficulty experienced upon no longer qualifying for food distribution from the World Food Program (food ratio cards are often reduced after a refugee’s first year in the settlement and sometimes are completely stopped after a few years). One refugee lamented, *“If not [for] this refugee life, I could be okay. Like when I was still in my country, I had no problem because I used to eat well and drink. These days, my problem is lack of what to eat and drink.”*

In this low resource setting, it is essential that one’s home be sufficient to provide shelter from the elements and that one has access to land suitable for planting food and keeping livestock. Many refugees complained their shelters were in disarray. They reported having no materials for repairing their shelters, and complained their land was too small to provide for their family and/or not proper for planting or keeping animals. As one participant explained:

"Look if someone comes to visit you, he can easily determine whether you have a good life. If you are getting food from your garden, have a goat or a cow and do not depend on what you work for as a daily survival means… But for us refugees, we depend on external sources of survival."

Many reminisced about times when the clinic distributed food and household items. They shared their desires to again obtain goods and services from the clinic, including: seeds, sugar, soy flour, roofing materials, school fees and books for their children, and employment opportunities.

Against the backdrop of daily struggles, participants also faced occasional threats to their safety. Some told of violent uprisings between ethnic groups preventing safe travel in the settlement and disrupting daily routines. This insecurity sometimes prevented patients from reaching the HIV Clinic. Participants recalled:

"There was a time when the Congolese had fought and blocked the road and were breaking the car screens that were passing their way. So, we had to pass the other side of the Nationals [Ugandan citizens] in [nearby town] and we had to go to [another nearby town] to get our drugs, because it was an emergency."

Efforts to obtain food, repair shelter, till the land and remain safe in this austere environment were all consuming for interviewees. Daily tasks required to meet survival needs were generally prioritized over other activities.

### When HIV testing trumped other priorities

Despite the need to focus efforts on day-to-day survival, the refugee participants spoke of specific circumstances which led them to access HIV testing. HIV testing occurred when: (a) priorities temporarily shifted away from daily survival-related tasks; (b) survival needs were met; and/or (c) barriers to HIV testing were reduced.

### (a) Priorities shifted

Refugees tested for HIV when life circumstances such as severe illness or exposure to HIV led to a temporary shift in priorities away from usual daily tasks. Many refugees described presenting to clinic for HIV testing and treatment as a last resort when they were already incapacitated by illness. They described changes in their body reflective of severe wasting and some told stories of people looking at them and bluntly telling them that they had AIDS. Patients told of having lack of energy and complete loss of appetite. One noted, *“Before I went for HIV testing, I used to get sick seriously. I used to have high fever and cough and finally I had to go for HIV testing.”* For some, testing occurred when significant illness limited the ability to meet daily survival needs, leaving no other option but to seek medical assistance. A study participant explained, *"When someone is sickly, [and] cannot do his or her work properly, it becomes easy for that person to be encouraged to go for testing."*

Study participants also temporarily shifted priorities toward testing when fearing they had been acutely exposed to HIV. Some refugees informed by family or friends that their partner was presumed to have engaged in high-risk sexual behavior opted for testing. As one participant explained, *"Like here in [the trading center in the center of the settlement], men exchange women like clothes. For example, if someone tells you: ‘I have seen your husband with the other infected lady,’ you fear and come for an HIV test."*

### (b) Survival needs met

When survival needs were temporarily met, HIV testing became a reasonable alternative. Weighed down by urgent requirements such as obtaining food, repairing shelter and maintaining security, anything that helped with these tasks made it easier for testing to occur. When the clinic provided incentives such as food and supplies, the burden to obtain these goods lessened and patients could temporarily rest from this obligation. *“During the time of our testing, people used to get food and many people got tested because of the food.”* Refugees also spoke of a willingness to test because of the potential for food incentives for those who tested positive and attended HIV clinic. One participant even told of “*cheating*” the system and registering multiple times under false identities to be able to repeatedly test for HIV and receive food supplies. This participant said people sold the food items to other refugees at a considerable profit. She explained:

“So some people used to cheat and get the food with those who are HIV positive…this is because there was money out of the food, because someone would steal the food given and get like 150,000.00 Ugandan shillings [approximately $60 U.S. dollars]. In fact for those who were expert in cheating, [they] would sell food up to 300,000.00 Ugandan schillings [approximately $120 U.S. dollars].”

### (c) Barriers reduced

Testing became a viable alternative when conditions shifted to reduce barriers to accessing testing. When HIV testing was offered free in Nakivale Refugee Settlement at the GIZ Clinic, and people no longer had to travel outside the settlement to access this service, many people were tested for HIV. A refugee explained, “*It is easy since we have the clinic within [Nakivale], not like if it would be in [nearby town]. Above all, we have the free treatment for the virus and we do not suffer the transportation problem going to [nearby town]. So testing is done free of charge, and we thank God for that.”* Testing became even more accessible when mobile testing units came to the neighborhoods within the refugee settlement. One participant recalled*, "It was the first days they started testing from here and people were very many and some of them could come back without being tested. After that, they started meeting people in their different zones and it is when I was tested."* By minimizing barriers to HIV testing, more refugees were able to utilize testing services.

### Promising interventions

**Figure 1:**
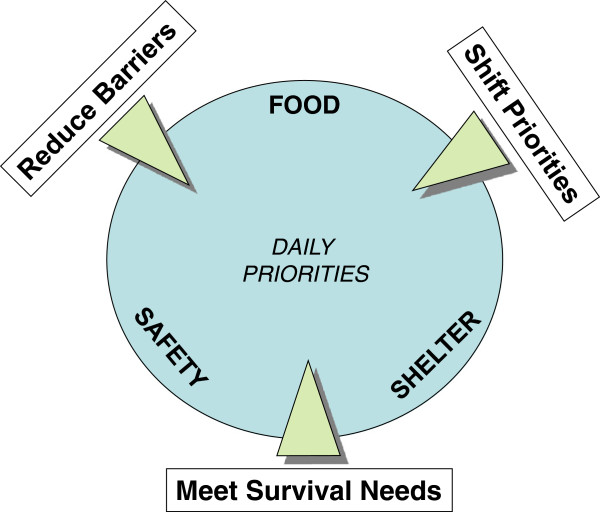
HIV Testing Interventions to Compete with Daily Priorities of Refugees; this figure depicts the daily priorities of refugees (food, safety and shelter) and three interventions which may effectively compete with these priorities (reduce barriers, shift priorities and meet survival needs).

Refugee and staff suggestions for interventions to increase utilization of testing services were consistent with this analytic framework (see Figure 1: HIV Testing Interventions to Compete with Daily Priorities of Refugees). When asked what would encourage testing, participants referenced educational campaigns and provision of food supplies to encourage shifting of priorities, as well as other services to meet survival needs and alleviate barriers to testing.

### (a) Interventions to shift priorities

Priorities can shift toward testing when the perceived benefit of testing is increased. Educational campaigns to decrease stigmatization and focus on the utility of ART and its impact on restoring strength and making physical work possible could be extremely effective in shifting priorities to encourage testing. In the words of study participants*, “If people can be sensitized about the spread, effects of the disease, and its related dangers if not treated, then people will come for the testing and treatment.”* A staff participant explained the importance of education and sensitization stating, “*…because some of them have a misconception about HIV. For example, the Somalis know that if you get HIV you automatically think of when to die.”*

Educational campaigns to encourage priority shifting toward HIV testing would likely be enhanced by using HIV positive refugees as peer educators to demonstrate the health and vitality of people living with HIV in this context. A number of participants shared this view:

"They can use people to sensitize others, they can use people like us who were very sick and people were counting our days to die and now we are fine. So if we can go out and tell the people that we are still living because of these drugs, people would be encouraged to take them."

“You sensitize your friends. Like sometimes when we are seated somewhere, say taking tea, where people are gathered, I tell them that, ‘you see I am taking my drugs,’ and they see that I am strong. ‘Do you know that I am still practicing my gymnastics? I can run for eight kilometers without even eating anything and I feel no side effects. Sometimes I even go out for hunting and I run after an antelope until I kill it!’”

By showing that HIV testing can improve overall functioning and ultimately survival, interventions may help refugees prioritize HIV testing over daily activities and increase utilization of testing services.

### (b) Interventions that meet survival needs

Respondents suggested meeting immediate survival needs with interventions that provide food and supplies to those who participate in testing. Programs have used these incentives in the past and study participants spoke of them as essential benefits to convince others to come to test. A participant advised*, “Serious sensitization is needed to remind people. Let there be some gift to give to the people who come for the testing. Many people would come without fear if the above are fulfilled.”*

Others suggested that HIV testing could be encouraged by providing special services for HIV positive patients attending clinic, stating that*, “When others will see that we are well cared for, they will be encouraged to voluntarily enroll for HIV testing and care."* Interviewees even went as far as to explain that when these advantages are not available to refugees with HIV/AIDS attending the clinic, others will not see a reason to come for testing.

“If there are different assistance offered to the people living with HIV, people can come for testing. I remember that before I could test for HIV, they could give some other feeding aids to the people. But now they no longer give us food. Others say that there is no need to test when there is nothing to gain after you have known that you have HIV.”

### (c) Interventions to alleviate barriers

Making HIV testing more accessible by eliminating barriers to testing is essential to increasing utilization of HIV testing in the refugee context. Refugees often travel a considerable distance to access services, expending considerable time and money. Many said that providing transportation to clinic would encourage people to test. Participants also described greater ease of accessing testing when the service became available within the settlement and especially when it was offered throughout the settlement in the different neighborhoods. When asked what interventions might encourage testing, one staff member responded, *“One of them could be reducing the distance they travel while coming here [to clinic] by introducing another health center near the people. This is to increase easy access.”* Similarly, another staff member answered, *“It is to provide transport and provision of services near the people. The distance has been a problem. Some want to test but they are limited by distance.”* To encourage staff to offer mobile testing, staff participants suggested that employees be offered incentives such as lunch and a small travel stipend.

Another barrier to HIV testing in this setting is the time it takes to attend a clinic appointment. The volume of patients waiting to be seen in the GIZ Clinic often meant that those desiring care had to arrive early in the morning and did not leave until late at night. This eliminated the possibility for them to accomplish any other tasks that day. Some suggested that streamlining the clinic process would result in more refugees accessing HIV testing and related care.

## Discussion

Nakivale refugee participants in this study prioritized daily survival needs such as obtaining food, maintaining shelter, cultivating land, and ensuring safety. Attending to these priorities meant other needs had to be postponed or foregone. In this setting, there is a tension between meeting the needs of immediate survival and meeting needs perceived as less urgent, such as preserving health. For the refugees interviewed, testing tended to occur when the tension temporarily eased, allowing priorities to shift. For some, priorities shifted when illness prevented involvement in normal activities, including obtaining food. For others, priorities shifted when immediate survival needs were temporarily met or when barriers to testing were reduced, allowing an opportunity to test. Understanding survival needs allows the development of targeted interventions to encourage priority shifting, in ways such as those outlined below. Qualitative research does not aim for generalizability; rather, the goal is to learn from the study participants and apply the knowledge gained to generate ideas for future studies.

Interventions that increase the perceived benefit of testing are likely to be a successful approach, because they will help HIV testing become an attractive competing priority compared with usual survival tasks. Presenting HIV testing and knowledge of HIV sero-status as advantageous could be done using educational campaigns as public health campaigns about HIV/AIDS have been shown to increase knowledge and to have a positive impact on health [41-43]. Highlighting the utility of medical therapy for those who are positive and the need to focus on safe sexual practices to decrease the likelihood of transmission for those who are negative, may incentivize testing and lead refugees to appreciate the advantage of knowing if they are HIV-infected or not. Enhancing the perceived benefit of testing could also be accomplished by providing material incentives at the time of testing as has been demonstrated in other settings in sub-Saharan Africa [44,45]. When offered a bar of soap or a small bag of maize, refugees may view having an HIV test as a reasonable priority over other competing demands.

Another potentially successful approach is to design interventions that help meet immediate survival needs. When able to disregard immediate needs, refugees will have the opportunity to use their time and energy to access available HIV testing services. This could be a temporary solution, seeking to provide respite for a short while, to encourage HIV testing during this time. Following the Partners in Health (PIH) model of care [46], which aims to alleviate the root causes of disease and provide long-term solutions to improve general health and well-being, interventions could focus on enhancing the quality of life of refugees rather than on providing a specific health service.

Perhaps the most promising and practical HIV testing interventions are those that focus on alleviating the barriers to testing. Given that HIV testing is provided free of charge, but that it is not conveniently located for many refugees, interventions designed to enhance accessibility are likely to yield increased utilization of services. A program that would build on this premise might be one that placed HIV counselors and testers in the waiting areas of health clinics in refugee settlements to offer HIV testing to people while they wait for clinical care, as has been demonstrated to be successful in other low resource settings in sub-Saharan Africa [47]. Though the current goal in many clinics is Provider-Initiated Testing and Counseling (PITC) for everyone seen by the clinician [1,5], the overwhelming number of patients seen daily by health care providers and subsequent time constraints make this impossible. By instead using lower-cost HIV counselors and testers to approach all-comers in the clinic waiting area, more refugees are likely to be offered testing and are more likely to utilize testing services.

If refugees are similar to other low-resource populations in Africa, a useful intervention might be mobile HIV testing units [30,34,44,45,48]. By bringing testing to remote communities, this makes accessing testing less time consuming and less costly. A more comprehensive but costly approach to mobile testing would be to offer home-based voluntary counseling and testing (HB-VCT) services to all refugees in the settlement. This type of program, though it would require significant resources, may have the biggest impact on helping more refugees test for HIV as it has been in non-refugee populations in sub-Saharan Africa [33,49-53]. Any HB-VCT program in a refugee setting to meet the medical needs of the refugees diagnosed as HIV-infected. Ideally this would include referral to an HIV clinic and arrangement of transportation to the clinic.

A number of limitations of this study should be considered. The study population included refugees diagnosed with HIV/AIDS attending ART Clinic and did not include people who had never tested for HIV/AIDS or people who were HIV-infected but not attending ART Clinic. This means a number of relevant topics could not be explored, including: the knowledge of HIV/AIDS among refugees who have never tested, awareness of the availability of HIV testing among refugees who have never tested, and self-risk assessment among refugees who have never tested. Since the study was conducted at the HIV clinic, and because refugees are often given land based on their country of origin, it is likely that specific country groups housed closer to clinic are over-represented in the HIV clinic and in the study. The interviews were conducted by a male from the Democratic Republic of the Congo. The fact that the interviewer was a male could have led some women to be timid in sharing information. Given the interviewer was from the Democratic Republic of the Congo, this also may have influenced the dialogue. The Democratic Republic of the Congo has been the site of ongoing war with complex interactions involving surrounding countries.

While these possibilities cannot be excluded, all of the refugees invited to join the study opted to participate and by report, most spoke candidly and outwardly expressed gratitude for the opportunity to tell their story. In addition, the conceptualization that followed from the analysis was based on the population studied in Nakivale Refugee Settlement. It is possible that in other refugee contexts the survival needs and daily priorities may differ.

## Conclusion

Focused efforts should continue to be made to encourage more refugees to test for HIV/AIDS. Grasping how HIV testing fits into the survival priorities of refugees, and recognizing circumstances that encourage refugees to opt for HIV testing, will inform development of effective interventions to help refugees access and utilize testing. Intervening to encourage priority shifting toward HIV testing, by helping to meet survival needs and by eliminating barriers to testing, will likely help more refugees use existing HIV testing services.

## Abbreviations

AIDS: acquired immune deficiency syndrome; ART: anti-retroviral therapy; GIZ: Gesellschaft für Internationale Zusammenarbeit; HIV: human immunodeficiency virus; MTI: Medical Teams International; UNAIDS: Joint United Nations Programme on HIV/AIDS; UNHCR: United Nations High Commissioner for Refugees; WHO: World Health Organization.

## Competing interests

The authors declare that they have no competing interests.

## Authors’ contributions

KNO and NCW conceived and designed the study. KNO obtained necessary approvals and led the efforts on implementation of data collection. ZMF conducted all of the interviews and KNO supervised data collection and data management. ZMF and KNO reviewed the data. KNO and SAH conducted the initial data analysis. KNO wrote the first draft of the manuscript. SAH and NCW participated in multiple manuscript revisions. ZMF contributed to minor manuscript revisions. KNO, NCW and SAH did the final manuscript revisions. KNO takes responsibility of the paper as a whole. All authors read and approved the final manuscript.

## Authors’ information

KNO is an emergency medicine physician at Brigham & Women’s Hospital/ Harvard Medical School, has her MPH from the Harvard School of Public Health, and studies HIV testing in refugees with the Medical Practice Evaluation Center at Massachusetts General Hospital. She is faculty at the Harvard Humanitarian Initiative. SAR is an emergency medicine physician in the Brigham & Women’s Hospital/ Harvard Medical School and is studying for her MPH at the Harvard School of Public Health. ZMF lives in Uganda, has his Masters of Arts in Development Studies and works for Bugema University. NCW is a medical anthropologist and Associate Professor in the Department of Psychiatry and the Department of Global Health and Social Medicine at Harvard Medical School. She conducts research on social and behavioral dimensions of HIV/AIDS treatment.
